# Comparative Study Between a Customized Bimodal Endoscope and a Benchtop Microscope for Quantitative Tissue Diagnosis

**DOI:** 10.3389/fonc.2022.881331

**Published:** 2022-05-12

**Authors:** Hussein Mehidine, Bertrand Devaux, Pascale Varlet, Darine Abi Haidar

**Affiliations:** ^1^ Université Paris-Saclay, CNRS/IN2P3, IJCLab, Orsay, France; ^2^ Université Paris Cité – Faculté de Médecine Paris Descartes, Paris, France; ^3^ Service de Neurochirurgie, Hôpital Lariboisière, Paris, France; ^4^ Department of Neurosurgery, GHU Paris Psychiatrie et Neuroscience, Paris, France; ^5^ Department of Neuropathology, GHU Paris-Psychiatrie et Neurosciences, Sainte-Anne Hospital, Paris, France; ^6^ IMA BRAIN, INSERM UMR S1266, Centre de Psychiatrie et de Neurosciences, Paris, France; ^7^ Université Paris Cité, IJCLab, Orsay, France

**Keywords:** fluorescence, brain tumors, endomicroscope, spectroscopy, fluorescence lifetime

## Abstract

Nowadays, surgical removal remains the standard method to treat brain tumors. During surgery, the neurosurgeon may encounter difficulties to delimitate tumor boundaries and the infiltrating areas as they have a similar visual appearance to adjacent healthy zones. These infiltrating residuals increase the tumor recurrence risk, which decreases the patient’s post-operation survival time. To help neurosurgeons improve the surgical act by accurately delimitating healthy from cancerous areas, our team is developing an intraoperative multimodal imaging tool. It consists of a two-photon fluorescence fibered endomicroscope that is intended to provide a fast, real-time, and reliable diagnosis information. In parallel to the instrumental development, a large optical database is currently under construction in order to characterize healthy and tumor brain tissues with their specific optical signature using multimodal analysis of the endogenous fluorescence. Our previous works show that this multimodal analysis could provide a reliable discrimination response between different tissue types based on several optical indicators. Here, our goal is to show that the two-photon fibered endomicroscope is able to provide, based on the same approved indicators in the tissue database, the same reliable response that could be used intraoperatively. We compared the spectrally resolved and time-resolved fluorescence signal, generated by our two-photon bimodal endoscope from 46 fresh brain tissue samples, with a similar signal provided by a standard reference benchtop multiphoton microscope that has been validated for tissue diagnosis. The higher excitation efficiency and collection ability of an endogenous fluorescence signal were shown for the endoscope setup. Similar molecular ratios and fluorescence lifetime distributions were extracted from the two compared setups. Spectral discrimination ability of the bimodal endoscope was validated. As a preliminary step before tackling multimodality, the ability of the developed bimodal fibered endoscope to excite and to collect efficiently as well as to provide a fast exploitable high-quality signal that is reliable to discriminate different types of human brain tissues was validated.

## Introduction

Despite being among the least frequently diagnosed cancers in the world (1), central nervous system (CNS) tumors occupy a high rank among the deadliest forms of cancer, especially the malignant types (2). Until today, total resection of the tumor mass is still the main therapy method adopted to treat the majority of brain tumor types, especially the malignant and infiltrating ones. Surgical operation is considered as the most critical and crucial step in brain tumor management. The main goal of such surgery is to remove the tumor mass as much as possible. The main challenge encountered by the surgeon is the ability to identify accurately the tumor margins as well the infiltrated cancerous cells all around in order to improve the operation quality and to reduce recurrence outcomes (3). These infiltrated margins present often similar appearances to the adjacent healthy tissues, and their removal is mandatory. If not, it will highly increase the recurrence rate of the tumor and its mortality after the operation (4). However, to ensure an optimal safe resection limit is reached and to confirm the success of the surgery, biopsy samples are extracted from the resection cavity for post-surgery histological analysis purposes. This analysis relies on a Hematoxylin and Eosin (H&E) staining of the extracted samples in order to obtain precise information on the nature of the sample tissue. Nevertheless, this analysis takes several days after the surgery to establish the final diagnosis. Relying on human judgment and the expertise of the anatomopathologist, this analysis is still the gold standard method to provide such information.

To address these issues and to improve the surgical gesture, several imaging techniques were proposed to guide the surgeon intraoperatively [i.e., intraoperative MRI (5), intraoperative ultrasound imaging (6)]. However, intraoperative retraction and tumor resection often result in brain shifts, making it challenging to assess the extent of resection in real time. In addition, the mentioned techniques lack reliability and high resolution.

In the last decades, several spectroscopic methods have been developed and added as an auxiliary to the intraoperative total resection process (7) such as fluorescence-guided surgery using 5-aminolevulinic acid (5-ALA). This technique allows direct fluorescence visualization and increases the rate and extent of high-grade glioma tumors (8). However, it has shown limited efficiency in identifying diffuse low-grade gliomas and micro-infiltration cases (9) and did not show a high specificity towards tumor cell regions (9, 10).

On the other side, label-free optical imaging techniques have emerged as novel tools that allow high-resolution and cross-sectional imaging methods such as Raman spectroscopy probes (11, 12), optical coherence tomography (OCT) (13, 14) and fluorescence imaging probes (15). These techniques could be used endoscopically to realize intraoperative high-speed imaging without using external markers. In addition, fluorescence microscopy and especially two-photon fluorescence (TPF) microscopy has shown a better ability than other techniques to discriminate healthy brain tissues from tumor ones. Unlike the conventional fluorescence microscopy, TPF emission requires the excitation of the fluorophore simultaneously by two photons with a higher wavelength than the emitted fluorescence light. Therefore, conventional blue laser light is replaced by near-infrared laser light, which corresponds to the photo-therapeutic window where water and blood have their lower absorption.

This excitation technique is able to provide high-resolution images that could compete with H&E images, which makes it more efficient as a non-invasive imaging technique for tumor site diagnosis (15, 16).

Thus, the endogenous fluorescence of human brain tissues (spectral emission intensity, molecular ratios, fluorescence lifetime, etc.) has been widely explored *ex vivo*, *in vivo*, and intraoperatively, in order to discriminate between tumoral, infiltrating, and healthy tissues (17–21). Tracking the alteration of the fluorescence signal has been proven to be an efficient method to monitor metabolism changes in cancerous tissues and to elaborate a specific metabolic signature for each tissue type (22–27).

In addition, another type of signal could be generated using two-photon excitation, the second harmonic generation (SHG). This signal is a non-linear optical coherent process that occurs when two incident photons that have the same frequency interact simultaneously with a non-linear structure, generating a single photon with exactly double the energy of the incident photons (half of the excitation wavelength). Unlike the fluorescence process, SHG does not involve absorption, which leads to the excitation of fluorescent molecules.

In biological specimens, non-centrosymmetrical structures, such as collagen fiber structures and muscle myosin, are a strong source of SHG signal. Therefore, SHG is considered as a strong indicator of vascularized structures related to tumor angiogenesis, where SHG levels have been observed to increase in tumor boundaries (28–30). It has been proved that SHG signal analysis, combined with two-photon fluorescence, is a powerful indicator for tumor boundaries delimitation as well for cell infiltration (29–31).

To this end, and in order to meet the surgeon needs, our team is developing a multimodal miniature two-photon fluorescence endomicroscope. This homemade developed setup will rely on analyzing the endogenous fluorescence of brain tissues with a multimodality of contrasts: two-photon fluorescence (TPF) imaging, SHG imaging and analysis, fluorescence lifetime (FLIM), and spectroscopic analysis (15, 32). This tool is intended to offer sub-cellular information on the examined tissue and a fast real-time diagnosis response intraoperatively.

In parallel, and using a TPF multimodal benchtop microscope, the establishment of a large optical tissue database was launched a few years ago (16, 25, 31, 33–38) through a collaboration with GHU Hospital centre in Paris. The purpose of building this database is to characterize all types of human brain tissues, whether tumoral or not, and to establish for each type a specific optical signature, based on several optical indicators extracted from their endogenous fluorescence emission. This database will be connected to our endomicroscope and will combine multimodal analysis (fluorescence and SHG detection correlated with fluorescence lifetime and metabolic ratio analysis) in order to establish, *via* fast discriminative algorithms, a fast and efficient diagnosis of cancerous activity in the examined regions.

In our previous works, we have shown the ability of TPF multimodal analysis to reveal brain tissue type (whether healthy or cancerous). Thus, we managed to discriminate cancerous from healthy tissues, with a high sensitivity and specificity relying on such multimodal analysis. We have demonstrated, through qualitative and quantitative image analysis, the ability of our technique to differentiate (1) high-grade and low-grade glioma (16), (2) grade I and grade II meningioma (31), and (3) secondary and primary brain tumor (33). In addition, our optical TPF + SHG images were confronted and correlated with their corresponding H&E histological images. “Blind” histological analysis was performed by our neuropathologist collaborators in GHU-Paris Hospital center on these images to discriminate healthy from tumor samples. Therefore, they were able to recognize the different tissue types with 88% sensitivity and 71% specificity (15), and without any previous training.

For the instrumental development of this endomicroscope, a detailed characterization of the endoscopic fiber has already approved its use for *in vivo* non-linear imaging (32, 39). Numerical and experimental studies have also been performed to characterize the pulse compression and its delivery through this endoscopic fiber as well as all optical related technical parameters (32, 40).

Actually, the image acquisition modality is not implemented yet, so our setup is considered as a bimodal endoscope since it is able to perform two modes of acquisitions: (1) spectral measurements of the fluorescence emission and (2) fluorescence lifetime acquisition of the excited fluorophores.

Nevertheless, our endomicroscope is intended to be an optical tool for brain surgery assistance and for differentiation of cancerous areas, and we have already demonstrated the capacity of our optical imaging and analysis techniques in discriminating cancerous areas through our previous works (15, 16, 25, 31) (33–38). Indeed, we carried out this study in order to prove the capacity of our endomicroscope to generate a reliable quantitative signal that will be used, by combining it with imaging modality, to diagnose tumor areas intraoperatively.

Therefore, the aim of this work is to investigate the ability of our endoscope to provide equivalent or similar results to those provided by the benchtop microscope, which is not adapted for clinical use. This benchtop multimodal microscope is considered as our standard reference for *in vitro* tissue diagnosis, especially since it is the same platform used for our tissue database construction (15, 16, 25).

Therefore, a comparison study between the bimodal response of our endoscope with the response of the benchtop multiphoton microscope platform is presented. We are investigating spectral data, molecular ratios, and fluorescence lifetime values extracted from both setups using a large cohort of fresh human brain samples.

## Materials and Methods

### Samples

Through a collaboration with GHU-Paris Hospital center and according to an approval from the GHU-Paris – University Paris Descartes Review Board (CPP Ile de France 3, S.C.3227), sample tissues were collected during surgery times from the Department of Neurosurgery and Neuropathology at the GHU-Paris Hospital. All methods and measurements were performed accordingly with the relevant guidelines and regulations of this approval, and informed consents were obtained from all patients. Forty-six fresh samples were extracted from thirty-six patients and transported to our laboratory to perform the measurements. **Table 1** shows the different types of tissues used in this study and their established diagnosis based on World Health Organization (WHO) classification of central nervous system tumors (41, 42), and confirmed by our neuropathologist collaborator.

**Table 1:** Summary of the sample cohort included in this study.

Tissue Type	Description	Number
Control	Epileptic tissues	14
		
Meningioma	Atypical meningioma	3
	Secretory meningioma	3
	Transitional meningioma	2
	Meningothelial meningioma	1
	Microcystic meningioma	1
	Rhabdoid meningioma	1
		
Glioma	Glioblastoma IDH-wild type	3
	Glioblastoma IDH-mutant	3
	Anaplastic oligo-astrocytoma	2
	Astrocytoma IDH-mutant	1
	Anaplastic astrocytoma	1
		
Metastasis	Lung carcinoma	3
	Adenocarcinoma	3
	Melanoma	3
	Thyroid carcinoma	2

### Benchtop Microscope

This platform consists of a Leica TCS SP8 multimodal multiphoton fluorescence benchtop microscope (Leica Microsystems, Wetzlar, Germany) controlled *via* Leica’s acquisition software, where its technical details were published elsewhere (15). Briefly, this platform includes a Ti : Sapphire tunable laser source from 690 nm to 1,040 nm (Mai Tai DeepSee, Spectra-Physics). The objective used is a 25× water-immersion (HCX IRAPO L 25X NA 0.95, Leica). A hybrid internal detector (HyD, Leica, Germany) was used for spectral measurements, while two other external non-descanned hybrid detectors (HyD-RLD, Leica Microsystems, Wetzler, Germany) were used for two-photon fluorescence, SHG, and FLIM imaging. The first one is dedicated to select the nicotinamide adenine dinucleotide (NADH) fluorescence signal when using 800 nm as excitation wavelength and SHG signal when using 890 nm, using a 448 ± 20 nm band-pass filter (Semrock, FF01-448/20-25). The second one is dedicated to select the flavins (FAD) fluorescence signal using a 520 ± 30 nm band-pass filter (Semrock FF01-520/35-25). For fluorescence lifetime imaging acquisition, a time-correlated single-photon counting module (PicoQuant TCSPC module, Berlin, Germany) was coupled with the two external hybrid detectors, which permit performing NADH and FAD lifetime imaging at 800 nm.

### Two-Photon Endoscope Setup

**Figure 1:**
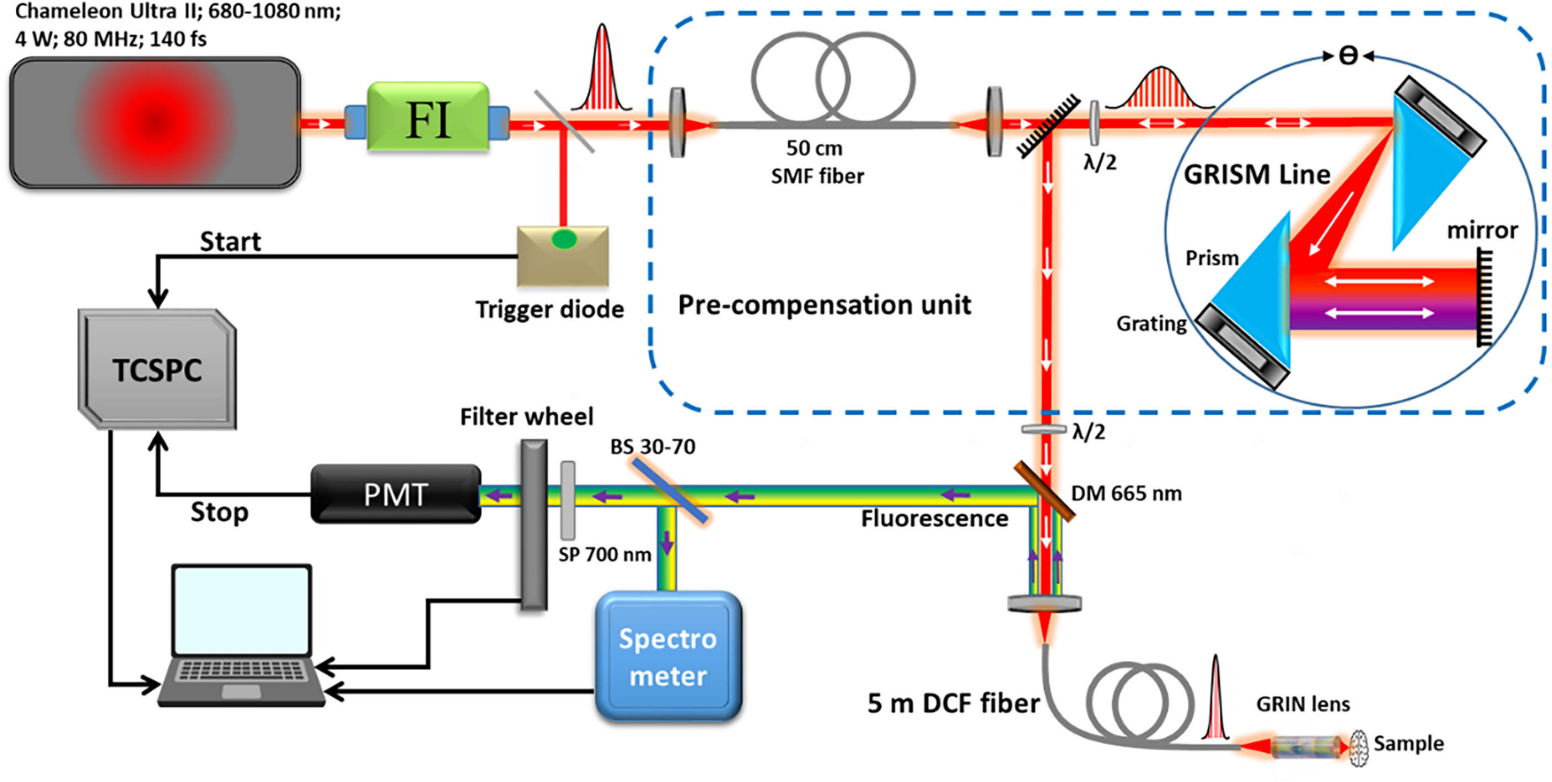
Illustration of the bimodal endoscope setup.

As presented in **Figure 1**, the bimodal endoscopic setup consists of a Ti : Sapphire laser source (Chameleon Ultra II, Coherent) tunable with an emission band of 680–1080 nm. This source is capable to generate pulses with a duration of 140 fs with a repetition rate of 80 MHz and an average power of 4 W at 800 nm. At the exit of the laser source, a Faraday Isolator (ISO-05-800-BB, Newport) was installed to protect the laser source from destabilizing feedback or actual damage from back-reflected photons. To compensate for the dispersion (second and third order) and the non-linear effects occurring inside the endoscopic fiber, a pre-compensation unit that consists of a polarization-maintaining single mode fiber (SMF) was used to broaden the pulse spectrum, as well as a GRISM (GRating+prISM) line stretcher (32, 40). The pulse duration at the output of the endoscopic fiber could be controlled and adjusted *via* this unit. Outgoing from the GRISM line, the laser beam is then coupled to a customized specific micro-structured double clad fiber (DCF). This fiber has a total outer diameter of 264 µm, and it was characterized and described in a previous work (32). It has a single mode central core to ensure the excitation with a diameter of a 6.4 µm and a numerical aperture of 0.097 (at 800 nm). It is surrounded by an air/silica micro-structured region with a diameter of 40 µm to separate the central core from the collecting inner cladding. The role of the latter is to collect the fluorescence signal with a numerical aperture of 0.27 and a diameter of 184 µm. The beam outgoing from the fiber is further focused using a gradient-index (GRIN) lens (GT-MO-080-018-AC-900-450, GRINTECH, Jena, Germany). At the exit of the fiber, the pulse duration width can be adjusted from 140 fs at the output of the laser cavity down to 40 fs. The captured fluorescence signal is then separated from the injected laser beam using a dichroic mirror, and then filtered through a short pass filter. A 70–30 beam splitter is also used to divide the signal into two parts: 70% directed toward a spectrometer (QE Pro, ocean insight) to analyze the spectra with a spectral resolution of 0.76 nm across a detection range from 200 to 1,000 nm. A minimum threshold of 100 recorded counts through the spectrometer at 1 s of acquisition time for an exploitable spectrum was fixed by our team. The remaining 30% are directed toward a PMT (PMA 182, PicoQuant, Germany) to analyze the fluorescence lifetime. The latter, before entering the PMT, passes through a motorized filter wheel, which comprises five band-pass filters dedicated to select the fluorescence signal of each molecule separately (488 ± 10 nm, 520 ± 10 nm, 580 ± 23 nm, 620 ± 13 nm, and 660 ± 13 nm). The instrument response function (IRF) of the TCSPC module was measured by recording the lifetime decay curve of the SHG signal of a urea crystal solution using 800 nm as excitation wavelength (Urea, U5128, Sigma Aldrich). The IRF corresponds to the time between the sending of the laser pulse by the laser cavity and its detection by the acquisition card. It is necessary to measure this electronic response of the measurement setup in order to be able to deconvolute the fluorescence decay curve and thus measure the fluorescence lifetime with more precision.

Spectral and lifetime measurements were conducted using 800 nm as excitation wavelength. The setup was optimized to deliver the maximum beam mean power and the shortest pulse duration at the output of the GRIN lens. With 1 W (25% of the maximum mean power delivered by our laser source), we managed to obtain 70 mW excitation mean power with 40 fs as pulse duration. The pulse duration was measured at the exit of the endoscopic fiber using an autocorrelator (Mini TPA/PD, APE, Germany).

## Data Analysis

### Spectral Data

Spectral data of the benchtop microscope were extracted *via* FIJI software and then it was plotted using a homemade developed Matlab software used in a previous work by our team (16). Using 800 nm as excitation wavelength, we are able to highlight the fluorescence emission of four endogenous fluorophores: NADH, FAD, lipopigments, and porphyrins. The same software was used to process the spectral data issued from the endoscopic setup. Through this software, the fluorescence emission of each fluorophore was represented by a Gaussian fit whose maximum wavelength and bandwidth follow the given values as shown in **Table 2**.

**Table 2:** Gaussian parameters used to fit the emission fluorescence spectra.

Fluorophores	Spectral bandwidth [nm]	Maximum emission range [nm]
NADH bound	40–48	443–445
NADH free	45–50	460–470
FAD	30–50	520–530
Lipopigments	0–180	570–600
Porphyrins I	0–10	615–630
Porphyrins II	0–10	675–690

Therefore, the integral proportion of each fluorophore was then calculated. It is defined by the ratio of the integral under the fitted fluorophore emission curve to the integral under the total fitted spectrum. Thus, we were able to extract and calculate three different molecular ratios following equations 1, 2, and 3 below:


(1) Redox ratio:FAD/(NADH+FAD)

(2) PN ratio:Porphyrins/NADH

(3) LP ratio:Lipopigments/Porphyrins

### Time-Resolved Data

The acquired fluorescence lifetime data were treated using the phasor approach. Contrary to the exponential fitting, this approach is a non-fitting technique. It consists of representing the fluorescence lifetime decay curve (FLDC) in a graphical view (43) where each FLDC is represented by a vector called “phasor” having its unique location in the phasor histogram. For the benchtop microscope FLIM data, the size of each image was reduced from 512 × 512 pixels to 16 × 16 pixels, adding each of the 32 × 32 pixels together to form a reduced pixel. Afterwards, each FLD of each reduced pixel was converted into two coordinates, Si (ω) and Gi (ω) in a Cartesian plot following equations 4 and 5:


(4) $${\text{S}}_{\text{i}}\left(\omega \right)=\frac{\int_{0}^{\infty }\text{I}(\text{t})\text{cos}\left(\text{ωt}\right)\text{dt}}{\int_{0}^{\infty }\text{I}(\text{t})\text{dt}}$$

(5) $${\text{G}}_{\text{i}}\left(\text{ω}\right)=\frac{\int_{0}^{\infty }\text{I}(\text{t})\text{sin}\left(\text{ωt}\right)\text{dt}}{\int_{0}^{\infty }\text{I}(\text{t})\text{dt}}$$

Si(ω) and Gi(ω) are respectively the *x* and *y* coordinates of the phasor corresponding to a reduced pixel “i” in the image, while *ω* is the laser repetition angular frequency related to the sampling period (Ts) and to the signal length (L) in equation 6 below:


(6) $$\text{ω}=\frac{2\text{π}}{\text{L}.{\text{T}}_{\text{s}}}$$

Adding to Si(ω) and Gi(ω), we also acquire a third parameter: the normalized integration under the FLDC. These three sets of numbers provided the phasor histogram with a FLIM image. Afterwards, the global phasor histogram grouping all phasor counts of all FLIM images of all samples is plotted. To calculate the fluorescence lifetime values corresponding to each molecule, the local maxima of the global phasor histogram is identified to draw a fitting line. The position of the two intersections of this fitting line with the universal circle are linked to the two fluorescence lifetimes values (43).

## Results

### Spectral Results

**Figure 2:**
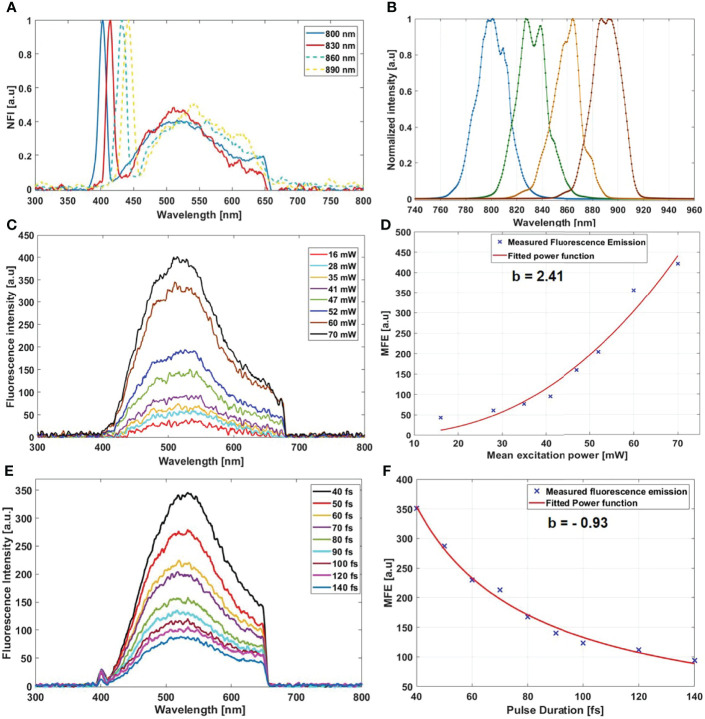
**(A)** Normalized fluorescence spectra acquired from a meningioma sample using 800, 830, 860, and 890 nm as excitation wavelength. **(B)** Shape of the excitation laser beam at the output of the endoscopic fiber at 800, 830, 860, and 890 nm. **(C)** Fluorescence spectra acquired from a control sample using several excitation mean power. **(D)** Variation of the maximum fluorescence emission (MFE) as a function of the mean excitation power. **(E)** Fluorescence spectra acquired using different pulse duration from a single region of interest of a GBM sample. **(F)** Variation of the maximum fluorescence emission (MFE) as a function of the laser beam pulse duration.

**Figure 3:**
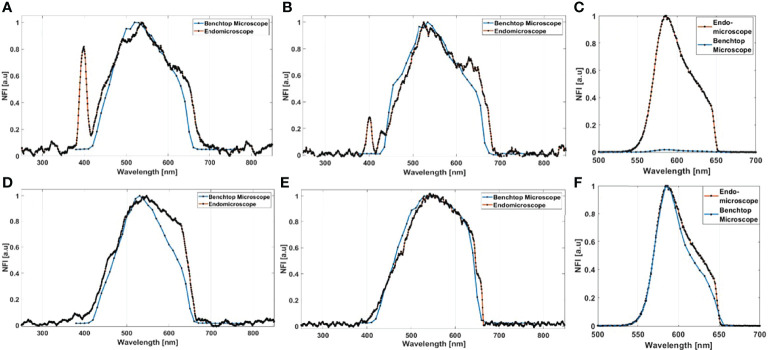
Normalized fluorescence intensity (NFI) spectra acquired from the bimodal fibered endoscope and from the benchtop microscope using 800-nm excitation for meningioma **(A)**, control **(B)**, metastasis **(D)**, and GBM samples **(E)**. **(C)** Comparison of the fluorescence maximum emission of Rhodamine-B solution, using 800 nm with 13 mW as excitation power, acquired from the benchtop microscope and from the fibered endoscope; the two acquired spectra were normalized to the peak intensity of the highest emission spectrum. **(F)** Comparison of the spectral shape of Rhodamine-B solution excited with the same power on the benchtop microscope and the fibered endoscope. The same spectra of **(C)** are presented, but each spectrum was normalized to its individual peak intensity.

**Figure 4:**
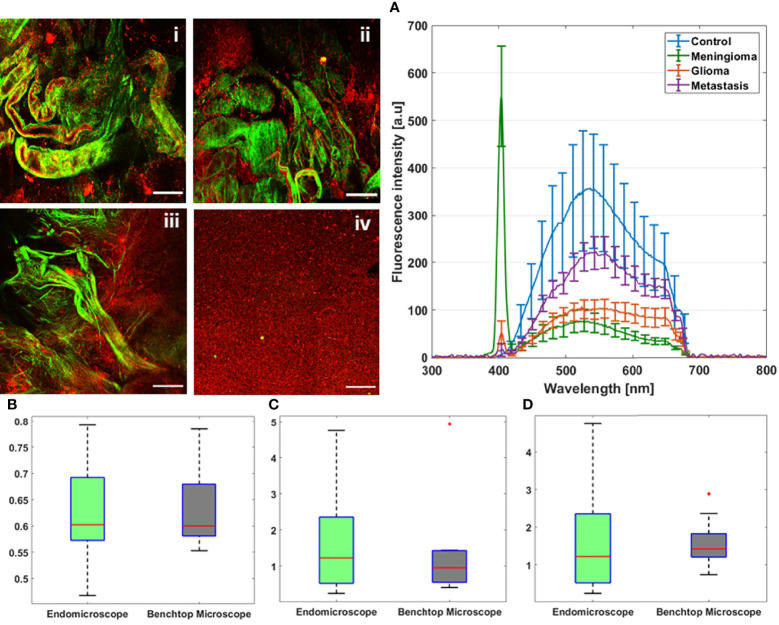
TPF and SHG images acquired at 890 nm as excitation wavelength through the multimodal benchtop microscope from GBM (i), meningioma (ii), metastasis (iii), and control (iv) sample. **(A)** Mean emission spectra of control, meningioma, glioma, and metastasis samples acquired using 800-nm excitation wavelength through the fibered endoscopic setup. Comparison between redox ratio **(B)**, PN ratio **(C)**, and LP ratio **(D)** derived from spectral measurements at 800-nm excitation through the benchtop microscope and the endoscopic setup. The values outside the box that are plotted in red “*” are considered as outliers.

Starting with the excitation and detection capabilities of the endoscopic setup, the spectral shape of the laser beam at the output of the endoscopic fiber was recorded using four different wavelengths: 800, 830, 860, and 890 nm. These spectra were plotted and are shown in **Figure 2B**. The spectral shape of the excitation laser beam did not change sharply with the wavelength, while each spectra was centered on its proper adjusted wavelength. To test the ability of using different excitation wavelengths and to confirm the excitation efficiency of our setup, we acquired the fluorescence spectra using the excitation wavelengths cited above from a meningioma sample presenting a high SHG emission. The four fluorescence spectra acquired under these excitation wavelengths are shown in **Figure 2A**. At 800, 830, 860, and 890 nm excitation wavelength, the SHG emission peak was centered at 402, 414, 432, and 442 nm, respectively. The measured shift was less than 4 nm for all spectra, which confirms the excellent tunable excitation efficiency of our endoscopic system. Once the collection efficiency through multiple excitation wavelengths is validated, the excitation power efficiency of the endoscopic setup should be demonstrated. Using 800 nm as excitation wavelength, a 70 mW as mean power can be reached at the exit of the endoscopic fiber. Despite being able to excite with such mean power value, we did not use it in our measurements, in order to avoid the risk of tissue heating or deterioration. Several spectral measurements were performed on a random control sample to figure out the minimum excitation power that we should use to get an exploitable spectrum. The spectra acquired using several excitation mean powers, with a pulse duration of 40 fs, were plotted and are shown in **Figure 2C**. Beyond 41 mW, the acquired fluorescence spectra exceed our fixed threshold. In addition, the variation of the maximum emission intensity as a function of the excitation power was determined. The fluorescence emission increased nearly 10 times from 16 to 70 mW. Plotted in **Figure 2D**, a fit power function was used to fit the variation of the fluorescence emission maximum as a function of the excitation power, where an exponent value *b* = 2.41 was found. Next, the pulse duration compression and its impact on the fluorescence emission was studied. From a random glioblastoma (GBM) tumor sample, and using 800 nm as excitation wavelength, the fluorescence spectra were acquired using several pulse durations from 40 fs, which is the minimal pulse duration reached by our system, up to 140 fs. **Figure 2E** shows the acquired spectra, using 41 mW as excitation mean power, for these different pulse durations. We can notice that the spectral shape is not affected as the pulse duration increases. The variation of the emitted intensity is positively correlated with the compressed pulse duration variation. This variation is shown in **Figure 2F**, where the maximum of the fluorescence intensity was plotted as a function of the pulse duration. Herein, we aim to prove and confirm the well-known inverse relation between the detected signal and the pulse duration. The fluorescence intensity is amplified four times when the pulse duration is compressed from 140 fs down to 40 fs. This variation was fitted into a power function where an exponent value *b* = −0.93 was found. Once the tunability, excitation power, and temporal pulse compression properties were validated, we conducted spectral measurements on the entire cohort of fresh samples. For each sample, we selected 3–4 regions of interest (ROI) depending on the sample size to perform local spectral acquisition using 800 nm as excitation wavelength. For all the measurement, the pulse duration was adjusted at the minimum reached value (40 fs) while the mean excitation power used was set at 41 mW. Afterwards, a comparison was performed between spectra of different tissue types acquired with the endoscope setup and with the benchtop microscope, as shown in **Figure 3**. Fluorescence spectra were recorded from a meningioma (**Figure 3A**), control (**Figure 3B**), metastasis (**Figure 3D**), and GBM (**Figure 3E**) fresh samples. Based on these acquired spectra, we can notice that the same spectral shape was obtained *via* the two different setups. At 800 nm excitation wavelength, the benchtop microscope is not able to detect SHG signal, due to the presence of a bandpass filter (448 + 20 nm) in front of the detector port, contrary to the fibered endoscope, which is able to detect SHG signal with a high sensitivity. We also noticed that the spectra acquired *via* the fibered endoscope were slightly wider in the right side corresponding to the porphyrin emission peak (650–680 nm), which is more noticeable in metastasis and GBM spectra. This shift is due to the higher quantum efficiency (90%) of the spectrometer used in the fibered endoscope setup than the hybrid internal detector of the benchtop microscope. Adding to that, a higher excitation mean power was needed to be used when using the benchtop microscope, which is not recommended for clinical use. **Figure 3C** presents the collected fluorescence spectra of 10 µl of Rhodamine-B solution (Rhodamine B, 83689, Sigma Aldrich) on the two different setups. The shown fluorescence spectra were acquired using 13 mW mean excitation power and 70 fs pulse duration for both setups and were then normalized to the peak intensity of the highest emission spectrum (the one extracted from the endoscope setup). The acquired signal from the fibered endoscope was much higher than the signal acquired from the benchtop microscope as our fibered endoscope setup collects the emitted signal more efficiently than the benchtop microscope setup. **Figure 3F** presents the same spectra of **Figure 3C**, but each spectrum is normalized to its maximum peak intensity. We can observe the same spectral shift observed approximately 630 nm in **Figures 3A**, **B** for tissues, indicating a more efficient fluorescence signal detection through the fibered endoscope. **Figure 4A** presents the mean spectra acquired through the fibered endoscope from all tissue types. All spectra were acquired at the same pulse duration and the same excitation mean power. Note that the error bars were divided by 3 for all types. Control tissues present higher fluorescence emission and a negligible SHG emission compared with other tumor types. Indeed, meningioma samples present a lower fluorescence emission but a higher SHG emission. These spectral observations were similar to those observed in the TPF and SHG images acquired from the benchtop microscope using an 890-nm excitation wavelength and presented in **Figure 4**i, ii, iii and iv. Looking to these images, we can see a clear TPF fluorescence image acquired from a control sample (**Figure 4**iv), while the meningioma image (**Figure 4**ii) is dominated by SHG signal resulting from the dense collagen fibrous structures. The same trend was observed in metastasis samples (**Figure 4**iii) with lower SHG signal and higher fluorescence signal. In the GBM sample (**Figure 4**i), we can see both fluorescence and SHG signals derived from the borders of large vessels. The last spectral characteristic was the molecular ratio extraction from the spectral fitting process. Using the fitting parameters shown in **Table 2**, all spectra acquired through the benchtop microscope and the fibered endoscope setup from all control samples were fitted in order to extract the emission contribution in the total spectrum of each excited molecule of NADH, FAD, lipopigments, and porphyrins. A comparison of the three molecular ratios extracted from both setups is shown in **Figure 4B** for redox ratio [FAD/(NADH+FAD)], **Figure 4C** for PN ratio (Porphyrins/NADH), and **Figure 4D** for LP ratio (Lipopigments/Porphyrins). Similar distributions were obtained for all three ratios with *p*-values of 0.77, 0.67, and 0.95, respectively. These values confirm the similarity of molecular ratios extracted from the benchtop microscope and the fibered endoscope. Therefore, we can validate the ability of our bimodal endoscope to extract valuable discriminative indicators between tumoral and healthy tissues.

### Time-Resolved Fluorescence Results

**Figure 5:**
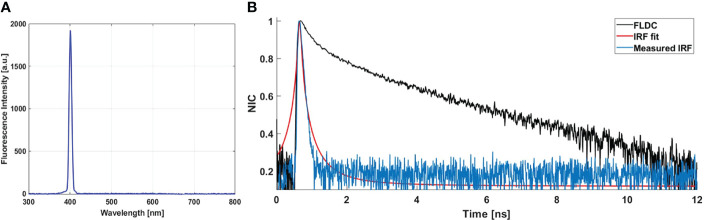
**(A)** SHG emission from urea crystal solution excited using 800 nm as excitation wavelength. **(B)** Measured IRF (blue curve) *via* the SHG emission spectra of urea crystals. Fitted curve of the IRF (red curve) *via* Lorentzian fit; fluorescence lifetime decay curve (FLDC, black curve) measured from a meningioma fresh sample.

**Figure 5A** shows the recorded spectrum of the urea crystal, used for the IRF measurement. These spectra show a unique peak centered at 402 nm with the same shift (2 nm) recorded for the SHG peak issued from meningioma samples (**Figure 2A**). The measured lifetime decay curve of this SHG signal, which corresponds to the IRF decay curve, is shown in **Figure 5B** (blue curve). It was compared to an FLDC issued from a fresh sample (black curve). The IRF decay curve was fitted *via* a Lorentzian fit (red curve, **Figure 5B**) where a full width at half maximum (FWHM) value of 88.2 ps was found. This value is negligible compared to a nanosecond-order fluorescence lifetime value measured by the fibered endoscope setup. This negligible value confirms the high system’s ability to resolve the fluorescence lifetime of the different endogenous fluorophores from the IRF.

**Figure 6:**
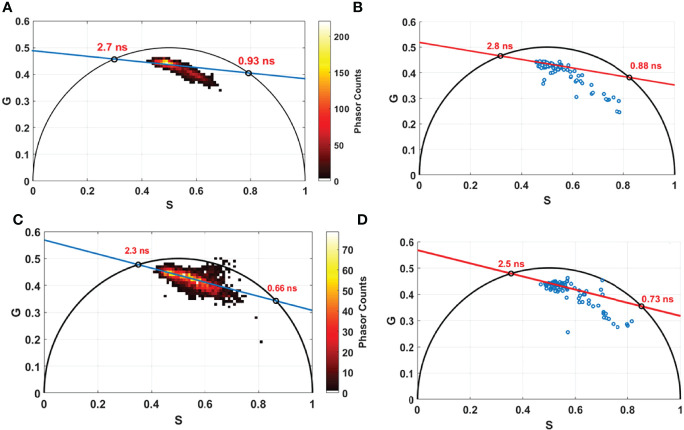
Phasor lifetime histogram acquired from the benchtop microscope **(A, C)** and from the fibered endoscope setup **(B, D)** for FAD **(A, B)** and for NADH (C, D). The two intersections of the fitting line with the universal circle in each figure corresponds to the long and the short lifetime in nanosecond (ns) of free and protein bound components of FAD and NADH.

In addition, we conducted lifetime measurements using an 800-nm excitation wavelength, on the bimodal endoscope for all control samples (14 samples) on several ROIs for each sample (same ROIs of spectral measurements). Similarly, FLIM measurements were conducted using the benchtop microscope, using 800 nm as excitation wavelength on the same samples. All collected data were treated and compared through the phasor lifetime method. **Figure 6** shows lifetime phasor histogram of FAD issued from the benchtop microscope (a, c) and from the fibered endoscope setup (b, d). The lifetime values obtained through the two setups were close to each other (2.7 ns and 2.8 ns for free FAD; 0.93 ns and 0.88 ns for protein-bound FAD). Likewise, NADH phasor histograms issued from the benchtop microscope and the fibered endoscope were compared and shown respectively in **Figures 6C, D**. The lifetime values obtained were close to each other but less than FAD lifetime values (2.3 ns and 2.5 ns for protein-bound NADH; 0.66 ns and 0.73 ns for free NADH). The obtained difference was 0.1 ns for FAD and 0.2 ns for NADH. This difference is still accepted regarding the fact that the number of the phasor counts in the benchtop microscope FLIM images is much higher than phasor counts acquired through the fibered endoscope. In addition, this difference could also be due to the different detectors and electronics used in both endoscope and benchtop microscope setups. Looking to the phasor cloud shape in both compared histograms, we can notice the similarity. In the NADH histogram, we can find several phasor counts outside the universal circle for both the benchtop microscope and the fibered endoscope in the same area.

## Discussion

In this work, we have presented a bimodal TPF endoscope dedicated to intraoperative imaging during a brain tumor surgery. We compared the bimodal response (spectral + lifetime) of this setup to a similar response generated by a standard reference setup, a multiphoton multimodal TPF benchtop microscope. This microscope was used in our previously published works concerning the discrimination of the different brain tissue nature (15, 16, 25). Therefore, we showed that our developed setup could deliver a high-quality signal, similar to that delivered by the standard reference, validating its efficiency and its capability to provide high-quality quantitative signal that could be used intraoperatively.

Nevertheless, the development of this type of instruments is not considered novel. In fact, several works have reported such setups with the ability to acquire high-quality *in vivo* TPF and SHG images from rat and mouse tissues (44–49). The team of F. Louradour discussed the addition of a FLIM modality to the TPF and SHG imaging capabilities (50, 51); this added modality allowed collecting additional information on the cellular energy metabolism during *in vivo* testing. However, most of the mentioned works performed their imaging tests on animal samples, and their studies were directed toward developing high-quality instrumental abilities. In addition, most of the reported setups are not intended, at least in the short term, for clinical applications in the operating room, since no tissue database specified to human brain and collected from endogenous fluorescence has been reported before. These instruments will not have the ability to offer to the surgeon a real-time diagnosis information on the examined tissue’s nature. Moreover, the application and the use of TPF and SHG imaging on a large number of human fresh samples were rarely reported before. Most of the reported *in vivo* imaging measurements were only performed on rat samples and without offering any multimodality perspective of analysis that could be applied later on human organs.

For that, we aim to develop an imaging tool that is able to combine multiple acquisitions with multimodal analysis of the acquired signal (spectral, temporal, and imaging) and that is connected to a large tissue database that gives it the ability to provide a reliable and real-time diagnosis information. Such tissue database is of significant importance for clinical use by neurosurgeons, and our team has already started to build such multimodal tissue database that aims to characterize all types of human brain tissues (healthy and tumors) in order establish a specific multimodal optical signature for each type (15, 16, 31, 33, 35–37).

However, having an instrument with advanced instrumental and technical abilities is also required. Our future setup should have the ability to generate a reliable signal that permits multimodal analysis.

For that, we are investigating in this work the ability of our setup to generate such signal. Several spectral and fluorescence lifetime measurements were performed on a large cohort of fresh human brain samples. Different excitation wavelengths, excitation mean powers, and pulse durations were used in order to investigate the differences between the response of the bimodal endoscope setup and the benchtop microscope one. The efficiency of exciting using several wavelengths is approved in **Figure 2A**. The shifted SHG peak between the different wavelengths showed that our excitation is suitable for a multiscale excitation. This ability to excite with different wavelengths allows our endoscope to excite efficiently using each of the four excited molecules, since they do not have a maximum absorption cross-section at the same wavelength. While NADH has a higher absorption cross-section at 800 nm, this cross-section remains very small at 890 nm where the porphyrins have a better cross-section than at 800 nm (52).

In addition, the collection efficiency of our instrument and its ability to excite with a high excitation power were examined. We found that exciting with 40 mW as mean power, corresponding to a pulse energy of 0.5 nJ at 80 MHz repetition rate, is sufficient to detect an exploitable spectrum. The obtained spectra using this mean excitation power (**Figure 2C**) are strong enough to be analyzed and to be used for image reconstruction. This efficient excitation will allow us to obtain high-quality TPF and SHG images and better signal-to-background noise ratio. The ability to deliver high excitation power will give us the capacity to better deal with solid tumors and to perform high-quality images on all types of brain tumor samples. The last examined property of the excitation process is the sub-fs pulse duration delivery, where we demonstrated that the fluorescence signal improved with the decrease of the pulse duration when compressing the laser pulse duration from 140 fs down to 40 fs. While the pulse duration is inversely proportional to the fluorophore’s absorption cross-section, shorter excitation pulses are requested also for superior image quality and consequently enhance the imaging depth and increase the number of recorded images per second (53).

Once the excitation wavelength, mean power, and pulse duration were adjusted, spectral and fluorescence lifetime measurements were conducted on our entire sample cohort through two different setups. These measurements show that both setups have similar efficiency of fluorophore emission detection, except for porphyrins. Indeed, our fibered endoscope offered a higher efficiency to collect low fluorescence signals and provided more precision to detect signals of low cross-section fluorophores at 800 nm, such as porphyrins. Therefore, this excellent efficiency allows using less mean power to excite our tissues, leading to the delivery of less pulse energy and, thus, less localized photo-damage. The fact that we use less optical elements in the detection path in the endoscope setup and a high-sensitivity and high-resolution spectrometer while exciting with a shorter pulse duration gives our fibered endoscope the ability to run fast acquisitions and to reach, in the future, a high imaging frame rate.

Indeed, the mean acquired spectra of all samples presented in **Figure 4A** have shown a remarkable difference of fluorescence and SHG emission between the different types of tissues. This difference is in harmony with what was reported in our past studies using the benchtop microscope (16, 35, 37). This comparison of spectral characteristic emission highlighted the performance of our bimodal endoscope and its ability to spectrally discriminate the different tissue types. This performance was confirmed through the TPF and SHG images acquired *via* the benchtop microscope. Furthermore, the similar distribution of the three molecular ratios, acquired from both setups, permits relying on these ratios extracted to establish a discriminative information connected to the metabolic activity of the tissue. These ratios were tested in our past studies *via* 3D discriminative algorithms and proved their power to diagnose, with a high specificity and sensitivity, different grades of tumors as well as healthy samples (15, 16).

As regards fluorescence lifetime analysis, the phasor technique was used to extract lifetime values from both benchtop microscope FLIM data and the decay curves acquired through the fibered endoscope. As a non-fitting technique, this method has the potential to simplify the analysis of FLIM images and to avoid the fitting errors associated with mono-exponential or bi-exponential fitting techniques (43). This method of analysis was used in our previous studies and managed to detect changes in glioma tumor based on molecular contribution and repartition of free and protein-bound state of FAD and NADH (16). The lifetime data acquired from both setups and the two main lifetime values of each molecule were close to each other. This similarity shows the potential of the fibered endoscope to rely on fluorescence lifetime analysis for discrimination studies.

Finally, the bimodality is the first step on the track of reaching a multimodal endomicroscope. In this work, the ability of our TPF bimodal fibered endoscope was proved to exploit the quantitative fluorescence signal of different human brain tissue types. This bimodal fibered endoscope revealed a low photo-damage and successfully provided a reliable and reproducible response through fast acquisition measurements (54). It is also able to generate low-damage ultra-short pulses with high mean power, all in the near-infrared window, the most efficient window for tissue excitation.

Furthermore, and through this work, the reliability of the quantitative response of this endoscope was demonstrated. This response was demonstrated by comparing it to a standard reference one based on previous published studies of our team.

After validating these mentioned requirements, the next step is to achieve the multimodality and to implement the qualitative aspect translated by the TPF and SHG image acquisition. For that, a customized endoscopic handheld imaging probe head, dedicated to intraoperative use, is under development. This imaging probe head consists of a miniature scanning system based on an electrothermally actuated microelectromechanical system (MEMS) mirror and has already been characterized to be utilized for *in vivo* imaging essay (55).

This endomicroscope will intervene during the surgery and after the removal of the tumor mass, which could be clearly defined with conventional pre-surgery imaging methods. However, our developed tool will help the surgeon to fully resect the tumor area as well the infiltrating zone residuals in order to achieve better maximal safe resection limits. It will offer a better visualization of the resection cavity at a cellular level, and may lead to extend the survival rate of the patient, which depends strongly on the extent of the resection.

Finally, a tool that gathers all the necessary assets for intraoperative use and that meets all technical and instrumental requirements needs to be implemented as a standard device in the operating room.

## Data Availability Statement

The raw data supporting the conclusions of this article will be made available by the authors, without undue reservation.

## Ethics Statement

The studies involving human participants were reviewed and approved by CPP Ile de France 3, S.C.3227. The patients/participants provided their written informed consent to participate in this study.

## Author Contributions

HM collected, analyzed the data, and wrote the manuscript. BD and PV provided samples and participated in writing. DAH designed the research project and the protocols for experiments, supervised the work, and participated in writing. Equally important, DAH provided necessary funds for the experiments and measurements criteria. All authors contributed to the article and approved the submitted version.

## Funding

This work is financially supported by ITMO Cancer AVIESAN (Alliance Nationale pour les Sciences de la Vie et de la Santé, National Alliance for Life Sciences and Health) within the framework of the Cancer Plan for MEVO and IMOP projects, by CNRS with “Défi instrumental” grant, by ligue nationale contre le cancer (LNCC) and the Institut National de Physique Nucléaire et de Physique des Particules (IN2P3). This work was done in the PIMPA Platform partly funded by the French program “Investissement d’Avenir” run by the “Agence Nationale pour la Recherche” (grant “Infrastructure d’avenir en Biologie Santé – ANR – 11-INBS-0006”).

## Conflict of Interest

The authors declare that the research was conducted in the absence of any commercial or financial relationships that could be construed as a potential conflict of interest.

## Publisher’s Note

All claims expressed in this article are solely those of the authors and do not necessarily represent those of their affiliated organizations, or those of the publisher, the editors and the reviewers. Any product that may be evaluated in this article, or claim that may be made by its manufacturer, is not guaranteed or endorsed by the publisher.

## References

[1] Bray F Ferlay J Soerjomataram I Siegel RL Torre LA Jemal A. Global Cancer Statistics 2018: GLOBOCAN Estimates of Incidence and Mortality Worldwide for 36 Cancers in 185 Countries. CA: A Cancer J Clin (2018) 68(6):394–424. doi: 10.3322/caac.21492 30207593

[2] Khodamoradi F Ghoncheh M Pakzad R Gandomani HS Salehiniya H. The Incidence and Mortality of Brain and Central Nervous System Cancer and Their Relationship With Human Development Index in the World. WCRJ (2017) 4(4):e985. doi: 10.32113/wcrj_201712_985

[3] Wilson T Karajannis M Harter D. Glioblastoma Multiforme: State of the Art and Future Therapeutics. Surg Neurol Int (2014) 5(1):64. doi: 10.4103/2152-7806.132138 24991467 PMC4078454

[4] Brown PD Maurer MJ Rummans TA Pollock BE Ballman KV Sloan JA. A Prospective Study of Quality of Life in Adults With Newly Diagnosed High-Grade Gliomas: The Impact of the Extent of Resection on Quality of Life and Survival. Neurosurgery (2005) 57(3):495–504. doi: 10.1227/01.NEU.0000170562.25335.C7 16145528

[5] Schulder M Carmel PW. Intraoperative Magnetic Resonance Imaging: Impact on Brain Tumor Surgery. Cancer Control (2003) 10(2):115–24. doi: 10.1177/107327480301000203 12712006

[6] Unsgaard G Rygh OM Selbekk T Müller TB Kolstad F Lindseth F. Intra-Operative 3D Ultrasound in Neurosurgery. Acta Neurochir (Wien) (2006) 148(3):235–53. doi: 10.1007/s00701-005-0688-y 16362178

[7] Lakomkin N Hadjipanayis CG. The Use of Spectroscopy Handheld Tools in Brain Tumor Surgery: Current Evidence and Techniques. Front Surg (2019) 6:30. doi: 10.3389/fsurg.2019.00030 31192217 PMC6548876

[8] Alston L Mahieu-Williame L Hebert M Kantapareddy P Meyronet D Rousseau D. Spectral Complexity of 5-ALA Induced PpIX Fluorescence in Guided Surgery: A Clinical Study Towards the Discrimination of Healthy Tissue and Margin Boundaries in High and Low Grade Gliomas. Biomed Opt. Express (2019) 10(5):2478. doi: 10.1364/BOE.10.002478 31149380 PMC6524587

[9] Tonn J-C. Fluorescence-Guided Resection of Malignant Gliomas Using 5-Aminolevulinic Acid. Clin Neurosurg (2008) 55:7. 19248665

[10] Stummer W Goetz C. Fluorescence-Guided Resection of Glioblastoma Multiforme by Using 5-Aminolevulinic Acid-Induced Porphyrins: A Prospective Study in 52 Consecutive Patients. J Neurosurg (2000) 93:11. doi: 10.3171/jns.2000.93.6.1003 11117842

[11] Desroches J Jermyn M Mok K Lemieux-Leduc C Mercier J St-Arnaud K. Characterization of a Raman Spectroscopy Probe System for Intraoperative Brain Tissue Classification. BioMed Opt Express (2015) 6(7):2380–97. doi: 10.1364/BOE.6.002380 PMC4505696 26203368

[12] Jermyn M Mok K Mercier J Desroches J Pichette J Saint-Arnaud K. Intraoperative Brain Cancer Detection With Raman Spectroscopy in Humans. Sci Transl Med (2015) 7(2740):274ra19. doi: 10.1126/scitranslmed.aaa2384 25673764

[13] Yashin KS Kiseleva EB Gubarkova EV Moiseev AA Kuznetsov SS Shilyagin PA. Cross-Polarization Optical Coherence Tomography for Brain Tumor Imaging. Front Oncol (2019) 9:201. doi: 10.3389/fonc.2019.00201 31001471 PMC6455095

[14] Pedrazzani M Breugnot J Rouaud-Tinguely P Cazalas M Davis A Bordes S. Comparison of Line-Field Confocal Optical Coherence Tomography Images With Histological Sections: Validation of a New Method for *In Vivo* and non-Invasive Quantification of Superficial Dermis Thickness. Skin Res Technol (2020) 26(3):398–404. doi: 10.1111/srt.12815 31799766

[15] Poulon F Pallud J Varlet P Zanello M Chretien F Dezamis E. Real-Time Brain Tumor Imaging With Endogenous Fluorophores: A Diagnosis Proof-of-Concept Study on Fresh Human Samples. Sci Rep (2018) 8(1):14888. doi: 10.1038/s41598-018-33134-2 30291269 PMC6173695

[16] Mehidine H Chalumeau A Poulon F Jamme F Varlet P Devaux B. Optical Signatures Derived From Deep UV to NIR Excitation Discriminates Healthy Samples From Low and High Grades Glioma. Sci Rep (2019) 9(1):8786. doi: 10.1038/s41598-019-45181-4 31217542 PMC6584506

[17] Toms SA Lin W-C Weil RJ Johnson MD Jansen ED Mahadevan-Jansen A. Intraoperative Optical Spectroscopy Identifies Infiltrating Glioma Margins With High Sensitivity. Operative Neurosurg (2005) 57:382–91. doi: 10.1227/01.NEU.000176855.39826.2D 16234690

[18] Yong WH Butte P. V. Pikul B. K. Jo J. A. Fang Q. Papaioannou T. Distinction of Brain Tissue, Low Grade and High Grade Glioma With Time-Resolved Fluorescence Spectroscopy. Front Biosci (2006) 11:1255–63. doi: 10.2741/1878 PMC2991156 16368511

[19] Kantelhardt SR Kalasauskas D König K Kim E Weinigel M Uchugonova A. *In Vivo* Multiphoton Tomography and Fluorescence Lifetime Imaging of Human Brain Tumor Tissue. J Neurooncol (2016) 127(3):473–82. doi: 10.1007/s11060-016-2062-8 26830089

[20] Marcu L French PMW Elson DS eds. Fluorescence Lifetime Spectroscopy and Imaging: Principles and Applications in Biomedical Diagnostics. Boca Raton: CRC Press/Taylor & Francis Group (2014).

[21] Butte PV Fang Q Jo JA Yong WH Pikul BK Black KL. Intraoperative Delineation of Primary Brain Tumors Using Time-Resolved Fluorescence Spectroscopy. J BioMed Opt (2010) 15(2):027008. doi: 10.1117/1.3374049 20459282 PMC4171753

[22] Croce AC Bottiroli G. Autofluorescence Spectroscopy and Imaging: A Tool for Biomedical Research and Diagnosis. Eur J Histochem (2014) 58(4):2461. doi: 10.4081/ejh.2014.2461 25578980 PMC4289852

[23] Croce AC Fiorani S. Locatelli D. Nano R. Ceroni M. Tancioni F. Diagnostic Potential of Autofluorescence for an Assisted Intraoperative Delineation of Glioblastoma Resection Margins. Photochem Photobiol (2003) 77(3):309–18. doi: 10.1562/0031-8655(2003)077<0309:dpoafa>2.0.co;2 12685660

[24] Li B-H Xie S-S. Autofluorescence Excitation-Emission Matrices for Diagnosis of Colonic Cancer. World J Gastroenterol (2005) 11(25):3931–4. doi: 10.3748/wjg.v11.i25.3931 PMC4504899 15991296

[25] Zanello M Poulon F Pallud J Varlet P Hamzeh H Abi Lahoud G. Multimodal Optical Analysis Discriminates Freshly Extracted Human Sample of Gliomas, Metastases and Meningiomas From Their Appropriate Controls. Sci Rep (2017) 7(1):41724. doi: 10.1038/srep41724 28150726 PMC5288720

[26] Skala M Ramanujam N. Multiphoton Redox Ratio Imaging for Metabolic Monitoring *In Vivo* . Methods Mol Biol (2010) 594:155–62. doi: 10.1007/978-1-60761-411-1_11 PMC2874879 20072916

[27] Skal MC Riching KM Gendron-Fitzpatrick A Eickhoff J Eliceiri KW White JG. *In Vivo* Multiphoton Microscopy of NADH and FAD Redox States, Fluorescence Lifetimes, and Cellular Morphology in Precancerous Epithelia. Proc Natl Acad Sci USA (2007) 104(49):19494–9. doi: 10.1073/pnas.0708425104 PMC2148317 18042710

[28] Thomas G van Voskuilen J Gerritsen HC Sterenborg HJCM. Advances and Challenges in Label-Free Nonlinear Optical Imaging Using Two-Photon Excitation Fluorescence and Second Harmonic Generation for Cancer Research. J Photochem Photobiol B Biol (2014) 141:128–38. doi: 10.1016/j.jphotobiol.2014.08.025 25463660

[29] Han J Daniel JC Pappas GD. Expression of Type VI Collagen During Glioblastoma Cell Invasion in Brain Tissue Cultures. Cancer Lett (1995) 88(2):127–32. doi: 10.1016/0304-3835(94)03627-U 7874684

[30] Pointer KB Clark PA Schroeder AB Salamat MS Eliceiri KW Kuo JS. Association of Collagen Architecture With Glioblastoma Patient Survival. J Neurosurg (2017) 126(6):1812–21. doi: 10.3171/2016.6.JNS152797 PMC5386834 27588592

[31] Zanello M Poulon F Varlet P Chretien F Andreiuolo F Pages M. Multimodal Optical Analysis of Meningioma and Comparison With Histopathology. J Biophoton (2017) 10(2):253–63. doi: 10.1002/jbio.201500251 26871683

[32] Ibrahim A Poulon F Habert R Lefort C Kudlinski A Haidar DA. Characterization of Fiber Ultrashort Pulse Delivery for Nonlinear Endomicroscopy. Opt. Express (2016) 24(12):12515. doi: 10.1364/OE.24.012515 27410272

[33] Poulon F Chalumeau A Jamme F Pallud J Varlet P Mehidine H. Multimodal Analysis of Central Nervous System Tumor Tissue Endogenous Fluorescence With Multiscale Excitation. Front Phys (2018) 6:109. doi: 10.3389/fphy.2018.00109

[34] Mehidine H Refregiers M. Jamme F. Varlet P. Juchaux M. Devaux M. Molecular Changes Tracking Through Multiscale Fluorescence Microscopy Differentiate Meningioma Grades and non-Tumoral Brain Tissues. Sci Rep (2021) 11(1):3816. doi: 10.1038/s41598-020-78678-4 33589651 PMC7884789

[35] Poulon F Mehidine H Juchaux M Varlet P Pallud J. Optical Properties, Spectral, and Lifetime Measurements of Central Nervous System Tumors in Humans. Sci Rep (2017) 7(1):13995. doi: 10.1038/s41598-017-14381-1 29070870 PMC5656602

[36] Mehidine H Sibai M Poulon F Pallud J Varlet P Zanello M. Multimodal Imaging to Explore Endogenous Fluorescence of Fresh and Fixed Human Healthy and Tumor Brain Tissues. J Biophotonics (2019) 12(3):e201800178. doi: 10.1002/jbio.201800178 30203459

[37] Sibai M Mehidine H Moawad EK Juchaux M Varlet P Devaux B. Comparison of Optically-Derived Biomarkers in Healthy and Brain Tumor Tissue Under One- and Two-Photon Excitation. J Biophotonics (2019) 12(11):e201900111. doi: 10.1002/jbio.201900111 31276313

[38] Haidar DA Leh B Zanello M Siebert R. Spectral and Lifetime Domain Measurements of Rat Brain Tumors. Biomed Optics Express (2015) 6(40):1219. doi: 10.1364/BOE.6.001219 PMC4399661 25909006

[39] Ibrahim A Poulon F Melouki F Zanello M Varlet P Habert R. Spectral and Fluorescence Lifetime Endoscopic System Using a Double-Clad Photonic Crystal Fiber. Opt Lett (2016) 41(22):5214. doi: 10.1364/OL.41.005214 27842096

[40] Lefort C Hamzeh H Louradour F Pain F Haidar DA. Characterization, Comparison, and Choice of a Commercial Double-Clad Fiber for Nonlinear Endomicroscopy. J Biomed Opt (2014) 19(7):76005. doi: 10.1117/1.JBO.19.7.076005 25003753

[41] Louis DN Perry A Wesseling P Brat DJ Cree IA Figarella-Branger D. The 2021 WHO Classification of Tumors of the Central Nervous System: A Summary. Neuro-Oncology (2021) 23(8):1231–51. doi: 10.1093/neuonc/noab106 PMC8328013 34185076

[42] Louis DN Perry A Reifenberger G von Deimling A Figarella-Branger D Cavenee WK. The 2016 World Health Organization Classification of Tumors of the Central Nervous System: A Summary. Acta Neuropathol (2016) 131(6):803–20. doi: 10.1007/s00401-016-1545-1 27157931

[43] Clayton AHA Hanley QS Verveer PJ. Graphical Representation and Multicomponent Analysis of Single-Frequency Fluorescence Lifetime Imaging Microscopy Data,”. J Microsc (2004) 213(1):1–5. doi: 10.1111/j.1365-2818.2004.01265.x 14678506

[44] Brown CM Rivera D. R. Pavlova I. Ouzounov D. G. Williams W. O. Mohanan S. *In Vivo* Imaging of Unstained Tissues Using a Compact and Flexible Multiphoton Microendoscope. J Biomed Opt (2012) 17(4):40505. doi: 10.1117/1.JBO.17.4.040505 PMC3382343 22559671

[45] Kim DY Hwang K Ahn J Seo YH Kim JB Lee S. Lissajous Scanning Two-Photon Endomicroscope for *In Vivo* Tissue Imaging. Sci Rep (2019) 9(1):3560. doi: 10.1038/s41598-019-38762-w 30837501 PMC6401070

[46] Allen J Pattie R Vance R Gu M. Fast Handheld Two-Photon fluorescence Microendoscope With a 475 µm ×475 µm field of View for In Vivo Imaging. Opt Lett (2008) 33:1333–35. doi: 10.1364/OL.33.001333 18552949

[47] Bao H Boussioutas A Jeremy R Russell S Gu M. Second Harmonic Generation Imaging *via* Nonlinear Endomicroscopy. Opt Express (2010) 18(2):1255. doi: 10.1364/OE.18.001255 20173949

[48] Zhang Y Akins ML Murari K. Xi J Li M Luby-Phelps J. A Compact Fiber-Optic SHG Scanning Endomicroscope and its Application to Visualize Cervical Remodeling During Pregnancy. Proc Natl Acad Sci (2012) 109(32):12878–83. doi: 10.1073/pnas.1121495109 PMC3420182 22826263

[49] Wu Y Leng Y Xi J Li X. Scanning All-Fiber-Optic Endomicroscopy System for 3D Nonlinear Optical Imaging of Biological Tissues. Opt. Express (2009) 17(10):7907. doi: 10.1364/OE.17.007907 19434122 PMC2696815

[50] Ducourthial G Leclerc P Mansuryan T Fabert M Brevier J Habert R. Development of a Real-Time Flexible Multiphoton Microendoscope for Label-Free Imaging in a Live Animal. Sci Rep (2015) 5(1):18303. doi: 10.1038/srep18303 26673905 PMC4682136

[51] Hage CH Leclerc P Brevier J Fabert M Le Nézet C Kudlinski A. Towards Two-Photon Excited Endogenous Fluorescence Lifetime Imaging Microendoscopy. Biomed Opt Express (2018) 9(1):142. doi: 10.1364/BOE.9.000142 29359093 PMC5772571

[52] Xu C Webb WW. Measurement of Two-Photon Excitation Cross Sections of Molecular Fluorophores With Data From 690 to 1050 Nm. J Opt Soc Am B (1996) 13(3):481. doi: 10.1364/JOSAB.13.000481

[53] Tang S Krasieva TB Chen Z Tempea G Tromberg BJ. Effect of Pulse Duration on Two-Photon Excited Fluorescence and Second Harmonic Generation in Nonlinear Optical Microscopy”. J Biomed Opt (2006) 11(2):020501. doi: 10.1117/1.2177676 16674172

[54] Sibai M Mehidine H Poulon F Ibrahim A Varlet P Juchaux J. The Impact of Compressed Femtosecond Laser Pulse Durations on Neuronal Tissue Used for Two-Photon Excitation Through an Endoscope. Sci Rep (2018) 8(1):11124. doi: 10.1038/s41598-018-29404-8 30042504 PMC6057889

[55] Mehidine H Li M Lendresse J-F Bouvet F Xie H Abi Haidar D. A Customized Two Photon Fluorescence Imaging Probe Based on 2D Scanning MEMS Mirror Including Electrothermal Two-Level-Ladder Dual S-Shaped Actuators. Micromachines (2020) 11(7):704. doi: 10.3390/mi11070704 PMC7408598 32708126

